# The neuroprotective effects of electrolyzed reduced water and its model water containing molecular hydrogen and Pt nanoparticles

**DOI:** 10.1186/1753-6561-5-S8-P69

**Published:** 2011-11-22

**Authors:** Hanxu Yan, Taichi Kashiwaki, Takeki Hamasaki, Tomoya Kinjo, Kiichiro Teruya, Shigeru Kabayama, Sanetaka Shirahata

**Affiliations:** 1 Graduate School of Systems Life Sciences, Kyushu University, 6-10-1 Hakozaki, Higashi-ku, Fukuoka 812-8581, Japan; 2 Department of Bioscience and Biotechnology, Faculty of Agriculture, Kyushu University, Fukuoka 812-8581, Japan; 3 Nihon Trim Co. Ltd., 1-8-34 Oyodonaka, Kita-ku, Osaka 531-0076, Japan

## Background

Human brain is the biggest energy consuming tissue in human body. Although it only represents 2% of the body weight, it receives 20% of total body oxygen consumption and 25% of total body glucose utilization. For that reason, brain is considered to be the most vulnerable part of human body against the reactive oxygen species (ROS), a by-product of aerobic respiration. Oxidative stress is directly related to a series of brain dysfunctional disease such as Alzheimer's disease, Parkinson's disease etc. Electrolyzed reduced water (ERW) is a functional drinking water containing a lot of molecular hydrogen and a small amount of platinum nanoparticles (Pt NPs, Table 1). ERW is known to scavenge ROS and protect DNA from oxidative damage [1]. We previously showed that ERW was capable of extending lifespan of *Caenorhabditis elegans* by scavenging ROS [2]. Molecular hydrogen could scavenge ROS and protected brain from oxidative stress [3]. Pt NPs are also a new type of multi-functional ROS scavenger [4].

**Table 1:** Characteristics of the water samples.The characteristics of water samples were determined immediately after the preparation of ERW. ERW, electrolyzed reduced water; CW, activated charcoal-treated water. The pH values were shown as average ± standard deviation (N = 5). The values of DH, DO and Pt NPs were shown the minimum and maximum values after 5 independent measurements.

	MQ (NaOH)	TI-200 ERW	TI-9000 CW	TI-9000 ERW
pH	11.3 ± 0.1	11.6 ± 0.1	7.9 ± 0.1	9.6 ± 0.2
Dissolved Hydrogen (mM)	0	0.2– 0.45	0	0.1– 0.25
Dissolved Oxygen (μM)	0	3.1– 78.1	0	0– 21.9
Pt NPs (nM)	0	0.5– 12.8	0	0– 3.6
Redox potential value (mV)	+ 350	-659	-	-

## Materials and methods

In this research, we used TI-200S ERW derived from 2 mM NaOH solution produced by a batch type electrolysis device and model waters containing molecular hydrogen and synthetic Pt NPs of 2-3 nm sizes as research models of ERW to examine the anti-oxidant capabilities of ERW on several kinds of neural cells such as PC12, N1E115, and serum free mouse embryo (SFME) cells. We pretreated the ERW and 200 μM H_2_O_2_ and examined the neuroprotective effects of ERW on PC12, N1E115 and SFME cells, using WST-8 method. We also examined the intracellular ROS scavenging effects of ERW on N1E115 cells after pretreated cells with ERW and H_2_O_2_ using DCFH-DA. We checked the protective effects of ERW on mitochondria and cytoplasm by Rh123 and Fuo-3 AM stain. We also examined the ATP production of SFME cells after pretreated with ERW and H_2_O_2_ by Bioluminescence Assay Kit. Finally, we used dissolved hydrogen (DH) and Pt NPs as research models to examine their neuroprotective effects.

## Results

**Figure 1:**
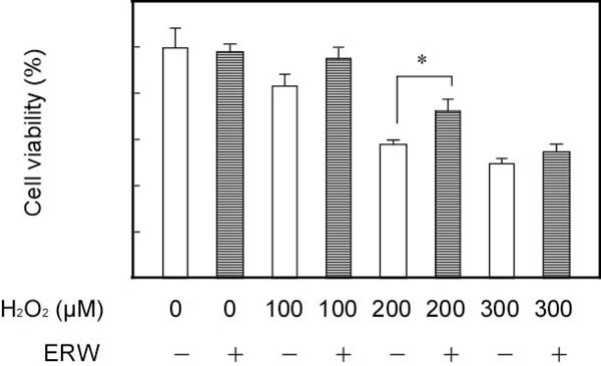
**Protective effect of ERW on H_2_O_2_-induced neuroblastoma N1E115 cells death.** Cells were treated with water samples (ERW and control ultrapure water with same pH with ERW) and 200 μM H_2_O_2_ for 24 h. Cell viabilities were assayed by WST-8 method. N=3, * p < 0.05.

ERW significantly reduced the cell death induced by H_2_O_2_ pretreatment (Figure 1). ERW also scavenged the intracellular ROS and prevented the decrease of mitochondrial membrane potential and ATP production induced by ROS. We also examined the neuroprotective effects of molecular hydrogen and Pt NPs and showed that both molecular hydrogen and Pt NPs contributed to the neuroprotective effects of ERW.

## Conclusion

The results suggest that ERW is beneficial for the prevention and alleviation of oxidative stress-induced human neurodegenerative diseases.
